# Total glucosides of paeony inhibit NLRP3/caspase-1/GSDMD-mediated inflammation and pyroptosis in C3H/HeJ mice with alopecia areata

**DOI:** 10.17305/bb.2024.10907

**Published:** 2024-09-11

**Authors:** Jingfang Zhang, Zhiquan Li, Kunpeng Liu, Xueyuan Du, Tao Yao, Jianzhou Ye

**Affiliations:** 1 Nanjing University of Traditional Chinese Medicine, Nanjing, China; 2 Department of Dermatology, Huiji District People’s Hospital, Zhengzhou, China; 3 Blood Purification Center of the First Affiliated Hospital of Henan University of Traditional Chinese Medicine, Zhengzhou, China; 4 College of Traditional Chinese Medicine, Henan University of Traditional Chinese Medicine, Zhengzhou, China; 5 Department of Dermatology, Yunnan Provincial Hospital of Traditional Chinese Medicine, Kunming, China

**Keywords:** Alopecia areata, total glucosides of paeony, NLRP3/caspase-1/Gasdermin D, pyroptosis, inflammation

## Abstract

One of the most prominent causes of alopecia areata (AA) is chronic inflammation of the hair follicles. Inhibiting cellular pyroptosis, a form of inflammatory programmed cell death, is crucial for reducing follicular inflammation in the skin. Total glucosides of paeony (TGP) possess anti-inflammatory properties across a broad range of illnesses. However, the role of TGP in AA and its relationship to pyroptosis remain unclear. A chronic unpredictable mild stress (CUMS) approach was used to create an AA mouse model. TGP suspension and MCC950 were administered to AA mice via gavage. HE staining, ELISA, immunohistochemistry, immunofluorescence, RT-qPCR, and Western blotting were performed to detect pathological changes in the skin and investigate the levels of inflammatory factors and pyroptosis-related proteins, as well as the potential mechanisms of TGP’s effects. TGP reduced hair loss, increased the number of hair follicles in skin tissues, and decreased inflammatory markers (IL-6, TNF-α, IL-18, and IL-1β) in AA mice. MCC950 significantly reduced the levels of NLRP3/caspase-1/Gasdermin D (GSDMD)-mediated pyroptosis-related proteins (NLRP3, ASC, caspase-1 p10, and GSDMD-N), as well as inflammatory factors. TGP markedly inhibited NLRP3/caspase-1/GSDMD-mediated cellular pyroptosis in a concentration-dependent manner. TGP suppresses the NLRP3/caspase-1/GSDMD signaling cascade in the skin tissues of AA mice, thereby reducing cellular pyroptosis and inflammation. TGP may be a potential therapeutic agent for AA.

## Introduction

Alopecia areata (AA) is a non-scarring autoimmune disease characterized by a chronic inflammatory response resulting from the destruction of the immune privilege of hair follicles [1], with a global prevalence of 2%. It can occur at any age, most commonly affecting young and middle-aged individuals, with no significant gender differences [2]. AA is prone to recurrence and increases the risk of other autoimmune diseases, such as hypothyroidism, systemic lupus erythematosus, vitiligo, and psoriasis [3, 4]. Clinically, AA is diagnosed using dermoscopy and histopathological examination [2], based on typical manifestations such as patchy hair loss on the scalp, or in severe cases, total hair loss [5], with inflammatory cells infiltrating the hair follicle epithelium and surrounding skin tissues [6]. AA significantly impacts patients’ quality of life and may lead to psychological disorders such as anxiety and depression [7, 8].

Currently, there are no treatments that can prevent or cure AA, and existing therapies focus on inhibiting or modulating the inflammatory invasion of hair follicles. These include topical treatments (e.g., clobetasol propionate, minoxidil, anthralin, and diphenylcyclopropenone), intralesional injections (e.g., triamcinolone acetonide and etanercept), phototherapy (psoralen plus ultraviolet A [PUVA]), and oral medications (e.g., JAK inhibitors and cyclosporine A). Although these therapies are effective, they are not recommended for prolonged use due to the risk of relapse and significant side effects [9]. Therefore, the search for new treatments with high efficacy and minimal side effects remains an important area of exploration.

Total glucosides of paeony (TGP) are active compounds extracted from the root of *Paeonia lactiflora* that have minimal side effects and possess immunomodulatory, anti-inflammatory, and hepatoprotective properties [10, 11]. Since 1998, TGP has been approved by the Chinese State Food and Drug Administration for the treatment of rheumatoid arthritis [12]. TGP has been shown to inhibit lymphocyte infiltration and suppress the activation of NLRP3 inflammasomes, thereby alleviating conditions such as dry skin syndrome [13]. It can also regulate the balance of proinflammatory and anti-inflammatory cytokines (e.g., IL-2/IL-7) to inhibit atopic dermatitis [14]. Furthermore, TGP has demonstrated clinical efficacy in treating immune-related skin diseases, such as psoriasis, systemic lupus erythematosus, and lichen planus [15–18]. Given these properties, we hypothesized that TGP may have therapeutic effects on AA. However, there is limited research on this topic, and the mechanism of TGP’s action in AA remains unclear.

TGP has been shown to suppress pyroptosis, thereby reducing inflammation and promoting tissue repair [19, 20]. Unlike apoptosis and necrosis, pyroptosis is a genetically regulated form of inflammatory cell death primarily driven by the activation of inflammasomes and inflammatory caspases. Excessive pyroptosis can lead to a range of autoinflammatory and autoimmune diseases [21, 22]. Inflammasomes activate caspase proteins, which regulate both classical and non-classical pyroptosis pathways [23]. In the classical pathway, caspase-1 plays a central role. Inflammasomes such as NLRP3, NLRC4, IPAF, and AIM2 activate and cleave pro-caspase-1 to generate active caspase-1, which then cleaves substrates such as Gasdermin D (GSDMD) and proinflammatory cytokine precursors (e.g., pro-IL-1β and pro-IL-18). This process leads to cell swelling, membrane rupture, and the release of inflammatory factors, triggering pyroptosis [24–28]. Previous research has shown that pyroptosis is a critical factor in autoimmune processes [29–31]. Thus, TGP may alleviate AA symptoms by reducing pyroptosis.

The NLRP3/caspase-1/GSDMD pathway is known to be a key mechanism initiating pyroptosis [32, 33], but its role in AA and whether TGP can ameliorate alopecia via this pathway has not been studied. Therefore, we investigated the effects of the NLRP3/caspase-1/GSDMD pathway on pyroptosis and inflammatory responses in the skin tissues of AA mice, as well as the potential mechanisms by which TGP modulates these inflammatory processes. This study aims to provide new insights into the treatment of autoimmune diseases using traditional Chinese medicine.

## Materials and methods

### Lab animals

Thirty-six healthy female C3H/HeJ mice, aged 6–8 weeks, were provided by Henan Kangda Laboratory Animal Co. (Zhengzhou, China). Before the experiment, the mice were acclimatized to the following conditions: a 12-h light/dark cycle, a room temperature of 25 ^∘^C, a relative humidity of 50%, and free access to food and water. The experiments adhered to the Guidelines for the Management and Use of Laboratory Animals, established by the Huiji District People’s Hospital of Zhengzhou Ethics Committee (No. KZ-20220509).

###  AA mice model construction

TGP (approval number: H20054521) was purchased from Liwah Pharmaceutical (Ningbo, China). The AA model was induced using a Chronic Unpredictable Mild Stress (CUMS) approach [34]. After seven days of acclimatization, the mice were randomly divided into the following groups: control (Control), AA model (AA), TGP low-dose (TGP-L, 0.1 g/kg/d), TGP medium-dose (TGP-M, 0.2 g/kg/d), TGP high-dose (TGP-H, 0.4 g/kg/d), and NLRP3 inhibitor (MCC950, 20 mg/kg). TGP and MCC950 were administered once daily via gavage at noon. Except for the control group, each set of six mice underwent a 21-day CUMS experiment, with seven different stimuli applied weekly, in a random order (Table 1). The mice were sacrificed on the 22nd day. The seven stimuli were: circadian reversal, 24-h fasting of food and water, 3 h of noise stimulation, an overnight tilted cage (8 h), a 3-min swim in 4 ^∘^C water, 1 min of tail suspension, and 15 min of horizontal electric shock at 100 Hz.

**Table 1:** The arrangement of the seven stimuli that C3H/HeJ mice were exposed to over 21 days

**Days**	**Simulation methods**
Day 1, 10, 21	Tilted cage overnight for 8 hours
Day 2, 11, 18	Swim for 3 minutes in icy water at 4 ^∘^C
Day 3, 14, 16	Water and food fasting for 24 hours
Day 4, 9, 19	Horizontal electric shock at 100 Hz for 15 minutes
Day 5, 12, 15	Noise stimulation for 3 hours
Day 6, 8, 17	Tail suspension for 1 minutes
Day 7, 13, 20	Circadian reversal

### Skin tissue sample preparation

Mice were anesthetized with 1% pentobarbital sodium (40 mg/kg) intraperitoneally. Blood was collected via eye removal, allowed to stand at 37 ^∘^C for 1 h, and then centrifuged at 4 ^∘^C for 10 min at 3000 r/min. The serum was transferred to EP tubes and stored at −80 ^∘^C. The skin from the backs of the euthanized mice was depilated and separated into sections. Some samples were preserved in 4% paraformaldehyde, while others were frozen at −80 ^∘^C.

### HE-staining

Fixed skin tissues were dehydrated, embedded, and sectioned (4 µm) using a microtome (RM2235, Leica, Germany). Sections were dewaxed with xylene and hydrated in an alcohol gradient. Hematoxylin (C0107, Beyotime, Shanghai, China) staining was performed for 15 min, followed by differentiation with 1% acidic alcohol (70% hydrochloric acid) for 30 s. After washing, sections were stained with 0.5% eosin (G1100, Solarbio, Beijing, China) for 3 min, dehydrated in an alcohol gradient, cleared with xylene, and sealed with neutral gum (G8590, Solarbio). Skin follicles and inflammatory infiltration were observed under a microscope.

### ELISA test

Samples were incubated at 37 ^∘^C with 50 µL of analytical buffer and 50 µL of sample for 2 h. The biotinylated antibody (100 µL) was added and incubated for 1 h at room temperature. After treatment at 25 ^∘^C for 1 h, 100 µL of TMB chromogenic reagent was added and incubated for 20 min in the dark. Finally, 50 µL of termination buffer was added. Absorbance was measured at 450 nm using a microplate reader, and content was estimated.

### RT-qPCR

Total RNA was extracted from skin tissues using the Trizol kit (DP424, TIANGEN, Beijing, China), and cDNA was synthesized using the Prime Script RT reagent kit (RR047 A, Takara, Tokyo, Japan). RT-qPCR was performed using SYBR (4309155, Applied Biosystems, DE, USA) on an ABI PRISM 7300 RT-PCR system (ABI, Carlsbad, CA, USA). β-actin was used as the internal control, and relative expression levels were calculated using 2^-ΔΔCt^. Primer sequences are as follows:

IL-6: F: 5′-GAGGTGAGTGCTTCCCCATC-3′; R: 5′-TTGCATCTGGCTTTGTTCGC-3′.TNF-α: F: 5′-ACTGATGAGAGGGAGGCCAT-3′; R: 5′-CCGTGGGTTGGACAGATGAA-3′.IL-18: F: 5′-CAGGCACTCCTAGCAGCTACA-3′; R: 5′-GCAGCGTGATTAAACCCAGG-3′.IL-1β: F: 5′-GGGGCGTCCTTCATATGTGT-3′; R: 5′-GGCAGCTCCTGTCTTGTAGG-3′.β-actin: F: 5′-GGCTTGCCACTCCCAAAGTA-3′; R: 5′-TCTGCGCTTCCTTTGTCCCC-3′.

### Western blot

Skin tissue samples were lysed in lysis buffer (20101ES60, Yeasen Biotechnology, Shanghai, China) at 4 ^∘^C for 30 min. The total protein was centrifuged, quantified, and heat-denatured. SDS-PAGE electrophoresis was used to separate the proteins, which were then transferred to a PVDF membrane and blocked for 15 min using a protein-free quick blocking buffer. The membrane was washed and incubated with primary and secondary antibodies. The primary antibodies used were rabbit anti-IL-6 (ab233706), TNF-α (ab183218), IL-18 (ab243091), IL-1β (ab254360), NLRP3 (ab263899), ASC (ab283684), caspase-1 p10 (AF4022), GSDMD (ab210070), GSDMD-N (ab215203), and the internal reference protein β-actin (ab8227), all purchased from Abcam. These were used at a concentration of 1:1000 and incubated overnight at 4 ^∘^C. The secondary antibody, goat anti-rabbit (1:2000, ab6721, Abcam), was incubated for 1 h. Protein bands were visualized using a chemiluminescence imaging system (5200, Tanon, Shanghai, China) and analyzed for grayscale values using ImageJ 1.8.0 software.

### Immunohistochemistry

Skin tissue sections (4 µm) were deparaffinized, hydrated, and subjected to antigen retrieval using citrate antigen repair solution (C1032, Solarbio). The sections were blocked with avidin/biotin blocking buffer (C-0005, HaoRan Biotech, Shanghai, China) at room temperature, followed by blocking with 3% BSA for 30 min. They were then incubated with rabbit anti-NLRP3 (1:50, ab270449, Abcam) and caspase-1 p10 (1:100, AF4022, Affinity) primary antibodies overnight at 4 ^∘^C, followed by incubation with goat anti-rabbit secondary antibody (1:500, ab150077, Abcam) at 37 ^∘^C for 1 h. The sections were stained using the streptavidin–horseradish peroxidase complex (OR03L, Sigma-Aldrich), incubated for 20 min at 37 ^∘^C, and developed with the Pierce DAB substrate kit (24002, Thermo Scientific, Waltham, MA, USA). Sections were counterstained with hematoxylin for 5 min, dehydrated through graded ethanol, cleared with xylene, and sealed with neutral gum. Microscopic examination and photos were taken.

### Immunofluorescence

BSA-blocked skin tissue sections were treated with rabbit anti-caspase-1 p10 (1:100, AF4022, Affinity) overnight at 4 ^∘^C, followed by incubation with a red fluorescent secondary antibody (1:500, ab150079, Abcam) for 1 h at room temperature. Sections were then stained with the Tunel staining kit (C1091, Beyotime) and counterstained with the DAPI kit (C1002, Beyotime). After sealing the sections, the number of caspase-1 p10 and Tunel-positive cells was counted using a fluorescence confocal microscope (Leica, Wetzlar, Germany).

### Data-based analysis

Statistical analyses and graphs were generated using GraphPad Prism 9.0 (GraphPad Inc., La Jolla, CA, USA). SPSS 26.0 software (SPSS Inc., Chicago, IL, USA) was used to assess the data for significance. Data were presented as mean ± standard deviation. ANOVA, *t*-tests, and chi-squared tests were used to compare groups. All experiments showed statistically significant results (*P < 0.05).

### Ethical statement

This study was approved by the Huiji District People’s Hospital of Zhengzhou Ethics Committee.

## Results

### TGP attenuates histopathologic skin damage in AA mice

**Figure 1.**
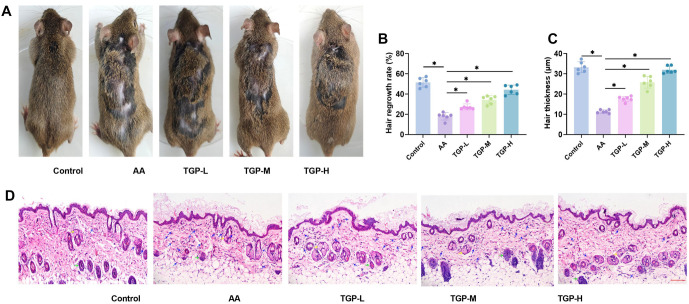
**TGP inhibited histopathologic skin deterioration in AA mice.** (A) Representative image of skin alopecia on the back of mice; (B and C) Changes in hair regeneration rate and hair thickness in mice; (D) HE staining results showed that compared to the control group, the number of hair follicles in the AA group was significantly reduced and a large number of inflammatory cell infiltration was observed in the surrounding area. Skin tissue damage gradually improved with the administration of TGP-L, TGP-M, and TGP-H. (20×, bar ═ 100 µm). *n* ═ 6, *P < 0.05. AA: Alopecia areata; TGP: Total glucosides of paeony; TGP-L: Low-dose group; TGP-M: Medium-dose group; TGP-H: High-dose group.

TGP attenuates histopathologic skin damage in AA mice. We randomly selected one mouse from each group of C3H/HeJ mice to compare hair shedding on their backs under various treatment conditions. Compared to the control group, mice in the AA model group showed significant hair loss over a large area of the back, with exposed skin displaying typical AA characteristics. After gavage with different concentrations of TGP to treat the AA mice, hair loss improved, the area of exposed skin decreased, and both hair regeneration rate and hair thickness increased: TGP-L>TGP-M>TGP-H (Figure 1A–1C). HE staining of skin tissue sections (Figure 1D) revealed that the AA model group had a significant reduction in sebaceous glands (yellow arrows) and hair follicles (green arrows), along with infiltration of inflammatory cells, primarily neutrophils and lymphocytes (blue arrows). In contrast, the control group had few or no inflammatory cells and an abundance of hair follicles. Compared to the AA group, the TGP-L group showed an increase in sebaceous glands and a reduction in inflammatory cell infiltration. The TGP-M group exhibited an increase in hair follicles, with hair growing into the site of sebaceous glands and encapsulated by inner root sheaths, and a reduction in inflammation-related cells. The TGP-H group had a significant decrease in inflammatory cells, along with abundant sebaceous glands and hair follicles. These results indicate that TGP gavage stimulated the production of hair follicles and reduced inflammation-induced damage in the skin tissues of AA mice, with the effect being dose-dependent.

**Figure 2.**
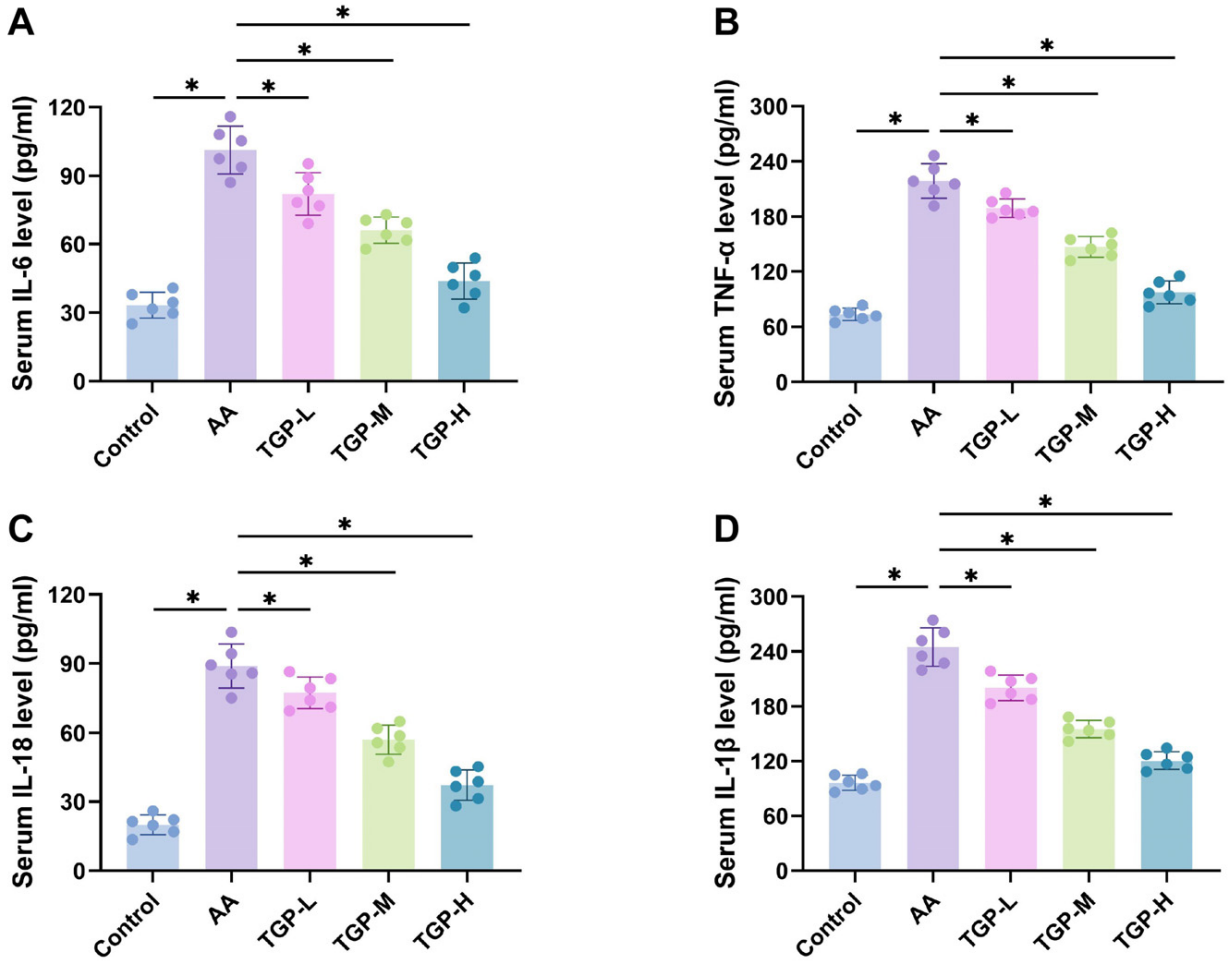
**TGP lowered serum levels of inflammatory factors in AA mice.** (A–D) TGP reduced the levels of inflammatory factors (IL-6, TNF-α, IL-18, and IL-1β) in AA mice’s serum, as assessed by ELISA. *n* ═ 6, *P < 0.05. AA: Alopecia areata; TGP: Total glucosides of paeony; TGP-L: Low-dose group; TGP-M: Medium-dose group; TGP-H: High-dose group.

### TGP lowers serum inflammatory factor levels in AA mice

The pathogenesis of AA involves the release of various inflammatory substances by cells, such as neutrophils and lymphocytes, which enhance the immune response and accelerate the progression of autoimmune diseases [6]. We used ELISA to measure the levels of inflammatory factors IL-6, TNF-α, IL-18, and IL-1β in the serum of different mouse groups. Figure 2A–2D shows that serum levels of all four inflammatory factors were significantly higher in the AA group compared to the control group. However, these levels gradually decreased with increasing concentrations of TGP. The results suggest that TGP gavage effectively reduced the levels of inflammatory factors in the serum of AA mice.

### TGP lowers inflammatory factor levels in skin tissues of AA mice

**Figure 3.**
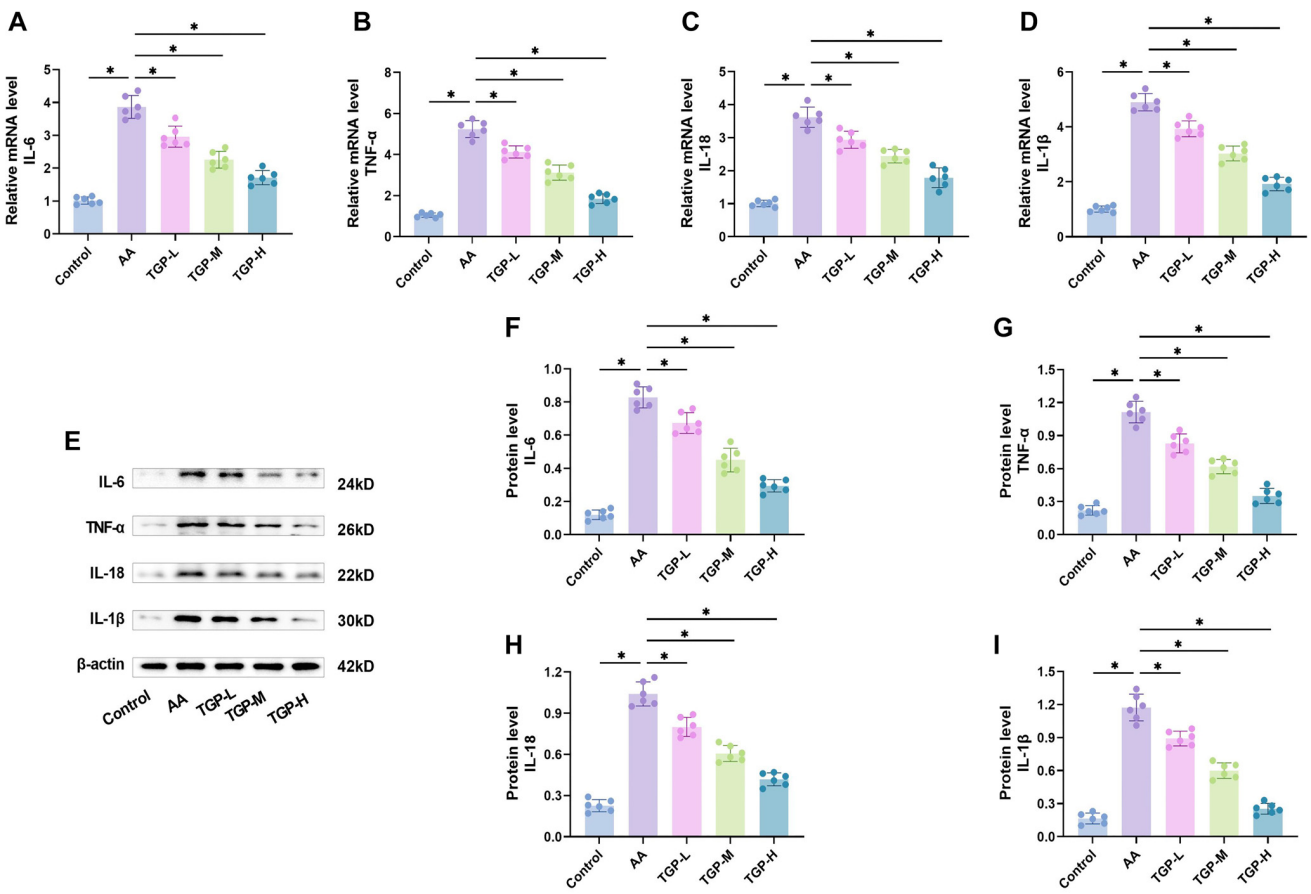
**TGP lowered inflammatory factor levels in the skin tissues of AA mice.** (A–D) TGP inhibited mRNA levels of IL-6, TNF-α, IL-18, and IL-1β in skin tissues in a dose-dependent manner, as measured by qRT-PCR; (E–I) TGP inhibited protein levels of IL-6, TNF-α, IL-18, and IL-1β in skin tissues in a dose-dependent manner, as demonstrated by Western blot analysis. *n* ═ 6, *P < 0.05. AA: Alopecia areata; TGP: Total glucosides of paeony; TGP-L: Low-dose group; TGP-M: Medium-dose group; TGP-H: High-dose group.

We next measured the expression of inflammatory factors in the skin tissues of each group of mice. RT-qPCR analysis of mRNA expression for IL-6, TNF-α, IL-18, and IL-1β in skin tissues was consistent with serum findings, with significantly higher levels in the AA group that decreased with increasing TGP concentration (Figure 3A–3D). Western blot analysis showed that the AA group had significantly higher levels of IL-6, TNF-α, IL-18, and IL-1β compared to the control group, but these levels decreased with higher concentrations of TGP treatment (Figure 3E–3I). These findings demonstrate that TGP reduced the inflammatory response in AA mice by inhibiting inflammatory elements in skin tissues.

### NLRP3/caspase-1/GSDMD-mediated pyroptosis in AA mice skin tissues

**Figure 4.**
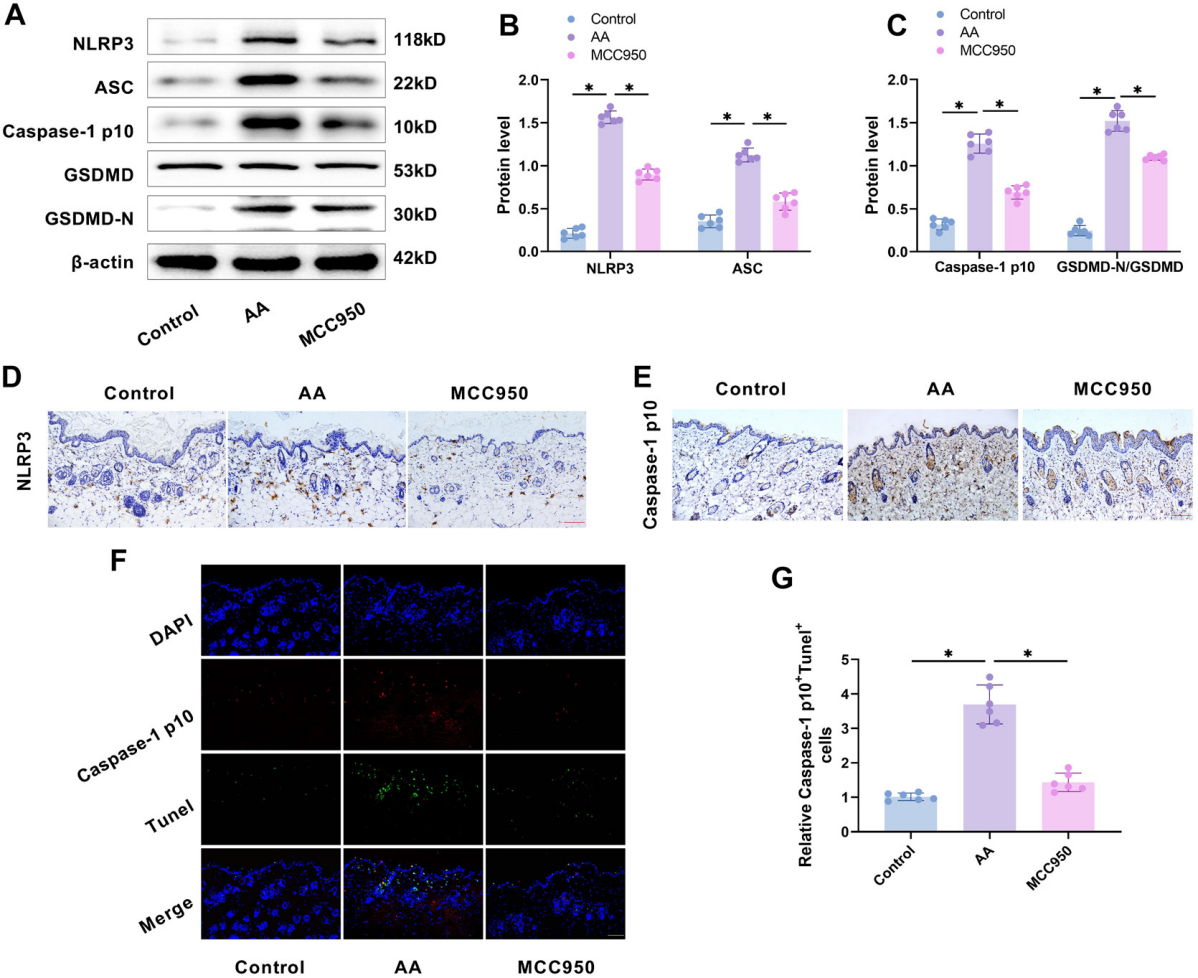
**NLRP3/caspase-1/GSDMD-mediated pyroptosis in the skin tissues of AA mice.** (A–C) Western blot analysis showed that treatment with MCC950 by gavage reduced the expression of pyroptosis-related proteins (NLRP3, ASC, caspase-1 p10, GSDMD, and GSDMD-N) in the skin tissues of AA mice; (D and E) Immunohistochemistry demonstrated that MCC950 gavage treatment decreased the expression of NLRP3 and caspase-1 p10 in skin tissues (20× magnification, scale bar ═ 100 µm); (F and G) Immunofluorescence data revealed that MCC950 gavage treatment reduced the intensity of caspase-1 p10 and Tunel fluorescence, and also decreased the number of positive cells. *n* ═ 6,*P < 0.05. AA: Alopecia areata; GSDMD: Gasdermin D.

Pyroptosis is a proinflammatory form of programmed cell death initiated by inflammasomes, resulting in robust inflammatory and immune responses [35]. Since TGP effectively reduced inflammation in AA mice, it is hypothesized that this effect involves the suppression of pyroptosis signaling. NLRP3 inflammasomes trigger the release of IL-1β, IL-18, and the formation of GSDMD pores by activating caspase-1, which leads to pyroptosis [36]. To test this, we administered the NLRP3 inhibitor MCC950 to AA mice via gavage and observed lesion development, along with changes in NLRP3/caspase-1/GSDMD-associated factors. Western blot analysis revealed that the levels of pyroptosis-related proteins (NLRP3, ASC, caspase-1, and GSDMD-N) were significantly higher in the AA group compared to the control group (Figure 4A–4C). Immunohistochemistry showed a marked increase in staining for NLRP3 and caspase-1 p10 in AA mouse sections compared to controls (Figure 4D and 4E), indicating activation of the NLRP3/caspase-1/GSDMD pathway. Double staining for caspase-1 p10 and Tunel revealed a higher number of caspase-1 p10 and Tunel-positive cells in the AA group compared to controls (Figure 4F and 4G), confirming that the NLRP3/caspase-1/GSDMD pathway mediated pyroptosis in AA mice. After gavaging AA mice with MCC950, we observed a significant decrease in the levels of pyroptosis-related proteins (NLRP3, ASC, caspase-1, and GSDMD) (Figure 4A–4E), along with fewer caspase-1 p10- and Tunel-positive cells (Figure 4F and 4G). These results suggest that MCC950 effectively inhibits NLRP3/caspase-1/GSDMD-mediated pyroptosis, confirming the role of pyroptosis in AA pathology.

### MCC950 attenuates inflammatory response in AA mice

**Figure 5.**
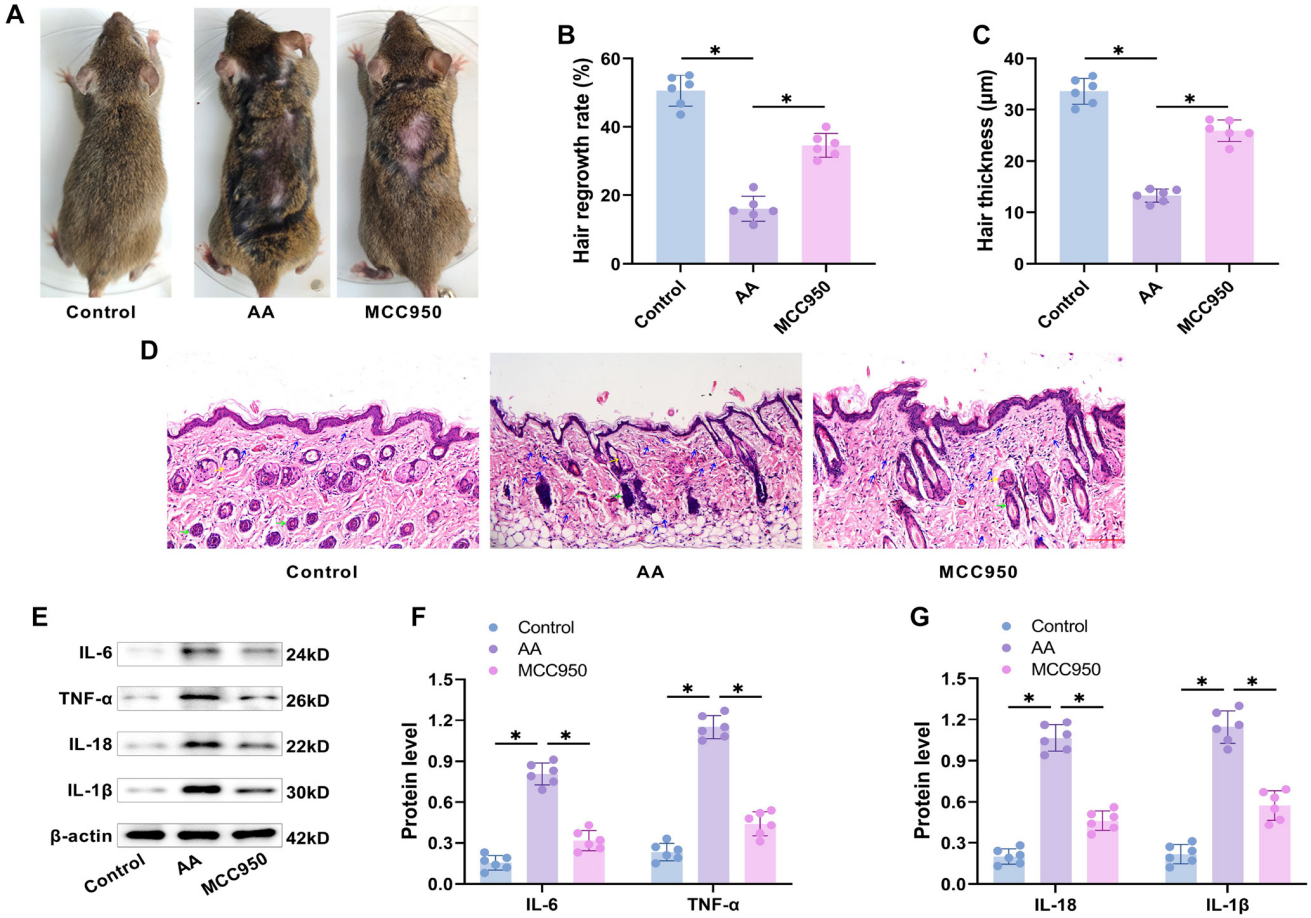
**MCC950 attenuated the inflammatory response in AA mice.** (A) Representative images of dorsal skin alopecia in mice; (B and C) Changes in hair regeneration rate and hair thickness in mice; (D) HE staining of skin tissue showed that treatment with MCC950 by gavage significantly improved skin tissue injury (20× magnification, scale bar ═ 100 µm); (E–G) Western blot analysis indicated that the MCC950-treated group had markedly lower levels of inflammatory markers in skin tissue compared to the AA group. *n* ═ 6,*P < 0.05. AA: Alopecia areata.

After gavaging MCC950, we examined its effect on inflammation via the NLRP3/caspase-1/GSDMD pathway in AA mice. The exposed skin area on the backs of MCC950-treated mice was significantly reduced compared to the AA group (Figure 5A), and both hair regeneration rate and hair thickness were significantly increased (Figure 5B and 5C). HE staining revealed a significant reduction in hair follicles and marked inflammatory cell infiltration in the AA group, whereas the MCC950 group showed an increase in hair follicles and a significant reduction in inflammatory cells (Figure 5D). Western blot analysis confirmed that MCC950 treatment dramatically reduced the expression of inflammatory factors (IL-6, TNF-α, IL-18, and IL-1β) in AA mouse skin tissues (Figure 5E–5G). These results suggest that MCC950 reduces inflammation in AA mice, resulting in improved hair loss. The study indicates that the NLRP3/caspase-1/GSDMD pathway promotes pyroptosis, which in turn exacerbates the inflammatory response in AA mice. In summary, we propose that TGP reduces inflammation in AA mice by inhibiting NLRP3/caspase-1/GSDMD-mediated pyroptosis.

### TGP inhibits NLRP3/caspase-1/GSDMD-mediated pyroptosis in skin tissues of AA mice

**Figure 6.**
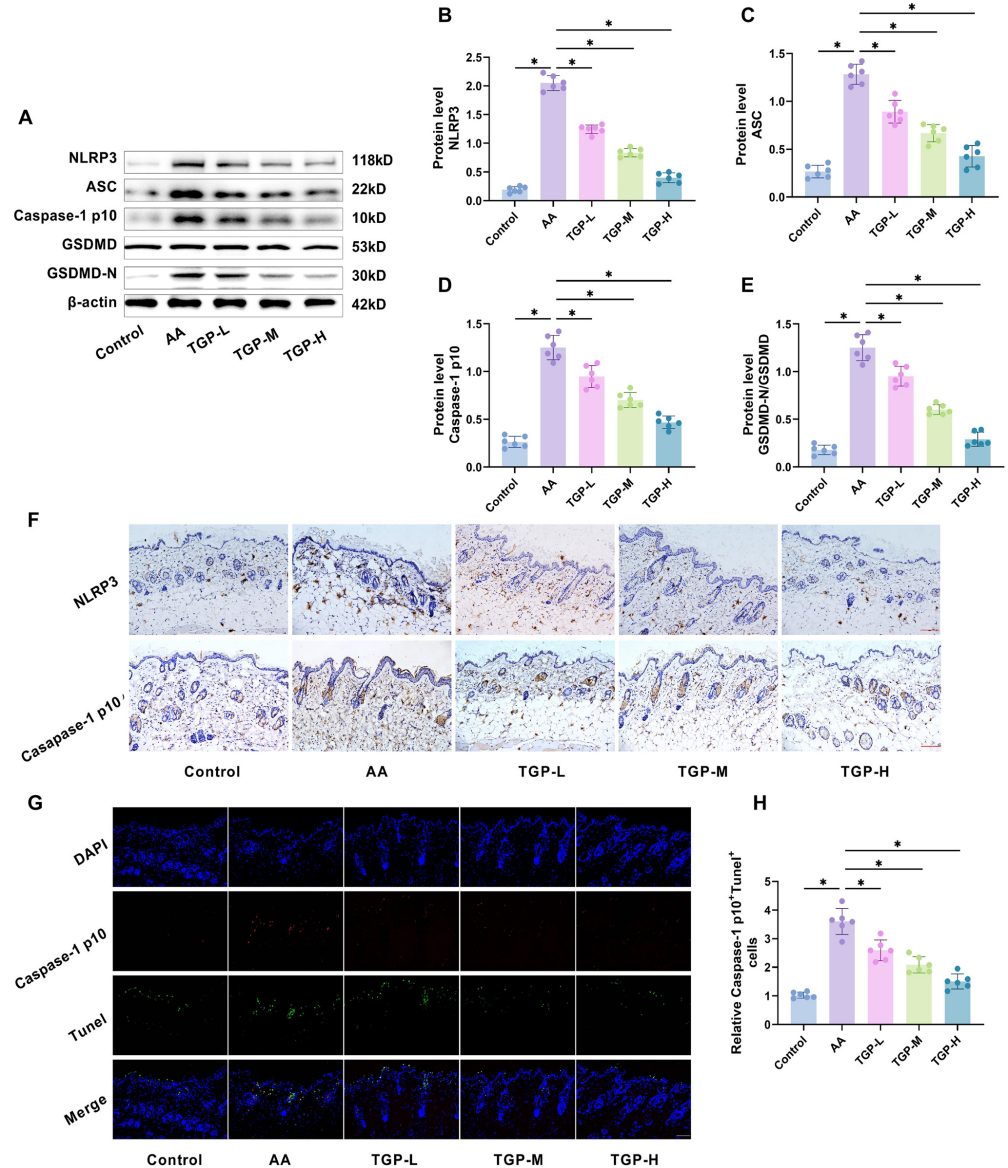
**TGP inhibited NLRP3/caspase-1/GSDMD-mediated pyroptosis in the skin tissues of AA mice.** (A–E) Western blot analysis showed that TGP reduced the levels of pyroptosis-related proteins (NLRP3, ASC, caspase-1 p10, GSDMD, and GSDMD-N) in the skin tissues of AA mice in a dose-dependent manner; (F) Immunohistochemistry revealed that TGP inhibited the expression of NLRP3 and caspase-1 p10 in the skin tissues in a dose-dependent manner (20× magnification, scale bar ═ 100 µm); (G and H) Immunofluorescence results demonstrated that TGP dose-dependently reduced the intensity of caspase-1 p10 and Tunel fluorescence, as well as the number of positive cells. (20×, bar ═ 100 µm). *n* ═ 6,*P < 0.05. AA: Alopecia areata; TGP: Total glucosides of paeony; TGP-L: Low-dose group; TGP-M: Medium-dose group; TGP-H: High-dose group; GSDMD: Gasdermin D.

To confirm this hypothesis, we investigated how TGP affects pyroptosis-related proteins and the number of caspase-1 p10- and Tunel-positive cells in the skin tissues of AA mice. Western blot analysis revealed that TGP gavage significantly inhibited the expression of NLRP3, ASC, caspase-1, GSDMD, and GSDMD-N in AA mouse skin tissues, with a concentration-dependent effect (Figure 6A–6E). Immunohistochemical results further confirmed that TGP inhibited NLRP3 and caspase-1 p10 levels in a concentration-dependent manner (Figure 6F). Additionally, as TGP concentration increased, the number of caspase-1 p10- and Tunel-positive cells in the skin tissues of AA mice decreased (Figure 6G and 6H). These findings suggest that TGP reduces NLRP3/caspase-1/GSDMD-mediated pyroptosis in AA mouse skin tissues, which may be a key mechanism underlying its anti-inflammatory effects.

## Discussion

AA is a genetic autoimmune illness that occurs when immune cells penetrate the hair follicle during the anagen phase when the hair follicle’s immune system is impaired [37]. During the hair cycle, the epithelium of the hair follicle maintains a zone of relative immune privilege, which promotes regular hair development [2]. An autoimmune reaction can disrupt the hair cycle, prematurely terminating growth, followed by follicular atrophy and alopecia [1]. Treatment for AA relies on decreasing autoimmunity by inhibiting the release of inflammatory factors and preventing immune cell infiltration. C3H/HeJ mice, which develop spontaneous alopecia due to autoimmune processes, are widely regarded as the best animal model for studying AA [38]. As a result, we chose C3H/HeJ mice to develop an AA model via CUMS induction. TGP, an anti-inflammatory herbal component, can alleviate skin pathology by regulating lymphocytes, reducing inflammation, and inhibiting the secretion of inflammatory molecules [39]. Our experimental results showed that as the dose of TGP increased, hair loss on the back of AA mice continued to improve, and the infiltration of inflammatory cells in the skin tissues gradually decreased. The expression levels of IL-6, TNF-α, IL-18, and IL-1β in the serum and skin tissues of the mice gradually decreased, consistent with previous reports. Furthermore, the CUMS method is frequently used to create depression models in mice, which induces significant psychological stress, resulting in anhedonia, weight loss, elevated corticosterone levels, thymic atrophy, and adrenal hypertrophy [40, 41]. TGP has also been shown to reduce neuroinflammation and provide antidepressant effects [42]. As a result, TGP may also reduce the psychological stress caused by CUMS in mice.

During the development of AA, lymphocytes, such as CD8+T and CD4+T rapidly infiltrate hair follicles. CD8+T cells target the inner section of the hair follicle, while CD4+T and NK cells concentrate near the outer root sheath, releasing pro-inflammatory cytokines, such as TNF-α and IL-6, which result in a breakdown of the follicle’s immune response [43, 44]. The conventional pyroptosis pathway is mediated by the NLRP3/caspase-1/GSDMD pathway. The formation of NLRP3 inflammasomes, composed of the innate immune receptor protein NLRP3, ASC, and caspase-1, is triggered by cellular stimulation, leading to the activation of caspase-1. This results in the cleavage of GSDMD, accompanied by the production of numerous inflammatory factors, including IL-1β, IL-18, and others [45]. The release of IL-18 and IL-1β into the extracellular space causes cell membrane pore formation, swelling, and rupture, triggering a cascade of amplified inflammatory responses. This can lead to the release of additional inflammatory factors, like TNF-α and IL-6, completing the pyroptosis process [46–49]. T lymphocytes can induce pyroptosis via the NLRP3 inflammatory programmed death pathway [50], while the activation of NLRP3 inflammasomes promotes the development of AA in C3H/HeJ mice [51]. Both NK and CD8+ T cells have been shown to kill Leishmania protozoa-infected cells, leading to the release of DAMPs, activation of NLRP3 inflammasomes, and IL-1β, which exacerbate skin ulceration [52]. These findings indicate that both T cells and NK cells located within or outside the hair follicle can activate the NLRP3/caspase-1/GSDMD pathway, accelerating the development of AA. Furthermore, NLRP3/caspase-1/GSDMD pathway-mediated pyroptosis can cause psychiatric disorders, such as depression [53], complicating AA treatment. Therefore, inhibiting the NLRP3/caspase-1/GSDMD pathway is highly beneficial for treating AA.

We found that MCC950 effectively suppressed the expression of pyroptosis-related proteins (NLRP3, ASC, caspase-1 p10, and GSDMD) and inflammatory factors (TNF-α, IL-6, IL-1β, and IL-18) in the skin tissues of AA mice. TGP, which had a similar effect to MCC950, also inhibited the expression of pyroptosis-related proteins and inflammatory factors in a concentration-dependent manner. The number of caspase-1 p10- and Tunel-positive cells in AA mice’s skin tissues significantly decreased following TGP treatment. These results suggest that TGP can substantially reduce inflammation in AA mice by inhibiting NLRP3/caspase-1/GSDMD-mediated pyroptosis. TGP may be a promising candidate for the treatment of AA, opening possibilities for the development of new AA medications. Furthermore, AA pathogenesis is caused by a complex interaction of genes and factors. Future research into other potential mechanisms by which TGP regulates AA, as well as toxicity evaluations and clinical trials, is required to advance the development of TGP-based Chinese medicinal preparations for clinical use.

## Conclusion

This research introduces a new potential therapy for AA in which TGP reduces the inflammatory response by modulating the NLRP3/caspase-1/GSDMD pathway, thereby improving AA symptoms. Different doses of TGP had varying effects on AA mice, with the highest concentration (0.4 g/kg/d) showing the strongest anti-inflammatory effect. While this study validated the effects of TGP in mice, future studies involving larger populations and clinical trials are necessary. It will also be important to determine the optimal drug delivery concentration, validate its efficacy for different areas and degrees of hair loss in patients, and design Chinese medicinal preparations that are widely applicable to AA patients and meet market standards. Furthermore, given that psychological stress and lifestyle habits (e.g., smoking, drinking, diet, and sleep) are significant factors in triggering or exacerbating AA [54], efforts should be made to raise awareness and educate AA patients and the general public about maintaining a healthy lifestyle.

## Data Availability

The data that support the findings of this study are available from the corresponding author, upon reasonable request.
